# METTL3 increases cisplatin chemosensitivity of cervical cancer cells via downregulation of the activity of RAGE

**DOI:** 10.1016/j.omto.2021.05.013

**Published:** 2021-06-04

**Authors:** Ruyi Li, Yizuo Song, Xin Chen, Man Chu, Zhi-wei Wang, Xueqiong Zhu

**Affiliations:** 1 Center for Uterine Cancer Diagnosis & Therapy Research of Zhejiang Province, Department of Obstetrics and Gynecology, The Second Affiliated Hospital of Wenzhou Medical University, Wenzhou 325027, Zhejiang, China

**Keywords:** METTL3, RAGE, cisplatin, chemosensitivity, cervical cancer, proliferation, apoptosis, tumorigenesis, MRP1, LRP1

## Abstract

Cervical cancer is the most common gynecologic malignancy worldwide. Methyltransferase-like 3 (METTL3) is involved in tumorigenesis; however, it is unclear whether METTL3 plays a potential role in regulating cisplatin (DDP) resistance. Therefore, the role of METTL3 in the regulation of cisplatin sensitivity in cervical cancer cells was determined. Our immunohistochemistry (IHC) data showed that METTL3 was highly expressed in para-cancerous compared with cervical cancer tissues. Furthermore, METTL3 overexpression inhibited viability and increased cisplatin sensitivity of cervical cancer cells *in vitro*. Overexpression of METTL3 inhibited tumor growth *in vivo*. IHC results showed that the receptor for advanced glycation and products (RAGE) had higher expression levels in cervical cancer tissues. RAGE downregulation increased cell sensitivity to cisplatin treatment. Moreover, the combination of FPS-ZM1 with cisplatin was more effective in inhibiting cell viability as compared with FPS-ZM1 or cisplatin only. Additionally, METTL3 reduced RAGE expression in cervical cancer cells. Overexpression of METTL3 downregulated RAGE expression and caused more sensitivity to cisplatin treatment in the SiHa-DDP cell line. METTL3 inhibited cell viability and increased apoptosis as well as enhanced the sensitivity of cisplatin via downregulating RAGE expression in cervical cancer cells.

## Graphical abstract

**Figure:**
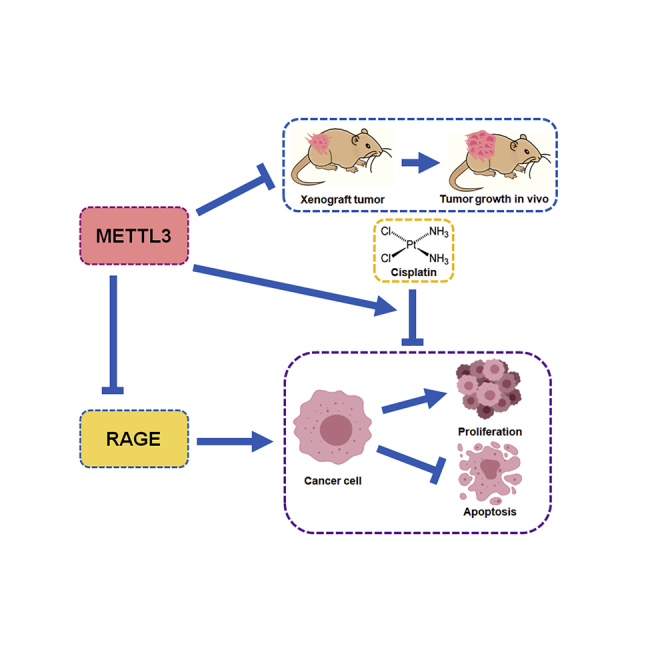

## Teaser

METTL3 is a novel tumor suppressor in cervical cancer tumorigenesis and progression. METTL3 inhibits the growth of cervical cancer cells both *in vitro* and *in vivo*. Moreover, METTL3 suppresses the activity of RAGE, thus increasing the cisplatin sensitivity in cervical cancer.

## Introduction

Cervical cancer is the most common gynecologic malignancy that threatens women’s health worldwide.^1^ Cisplatin is the main chemotherapeutic agent and is widely used to treat cervical cancer.^2^ However, acquired resistance to cisplatin often leads to poor outcomes and recurrence. Therefore, understanding the molecular mechanisms for chemotherapy resistance in cervical cancer and exploring new targeted therapies to overcome drug resistance are urgently needed.^3^

*N*^6^-methyladenosine (m6A) is the most common epitranscriptomic modification in mammalian mRNA.^4^ m6A methylation is reversible and regulated by adenosine methyltransferases^5^ and demethylases.^6^ Previous data have shown that the m6A level is significantly decreased in cervical cancer tissues and is tightly associated with cancer progression and poor survival.^7^ It is well known that m6A modification is catalyzed by a methyltransferase complex consisting of “writer” proteins such as methyltransferase-like 3 (METTL3) and METTL14.^5^ Genetic alterations of METTL3 could promote growth of cancer cells.^8^ Several studies regarding the role of METTL3 in cervical cancer have emerged.^9^, ^10^, ^11^, ^12^, ^13^ Most of these studies focus on the clinical relevance and prognostic value of METTL3 in cervical cancer. Our previous study revealed that quercetin potentiated the chemosensitivity to cisplatin in cervical cancer cells, along with the downregulation of METTL3 expression.^14^ However, the precise role of METTL3 in chemotherapy resistance of cancer cells and its downstream targets have not been fully understood.

The receptor for advanced glycation and products (RAGE) is a 35-kDa protein that belongs to the immunoglobulin superfamily.^15^ RAGE is highly expressed in many tissues of the developing embryo, but this expression is decreased in almost all tissues.^16^ Several studies have shown that RAGE signaling contributes to chronic inflammation, impaired cell communication, as well as aberrant activation of cell survival pathways, thus promoting tumorigenesis.^17^, ^18^, ^19^ Our team previously reported that RAGE promoted cell proliferation and inhibited apoptosis in cervical cancer cells.^20^ Nevertheless, whether RAGE is also closely related to cisplatin sensitivity of cervical cancer cells has not been explored yet.

In this study, the expression levels of METTL3 and RAGE were detected in both cervical cancer cells and tissues. The effects of METTL3 and RAGE on growth and cisplatin sensitivity were investigated in cervical cancer cells. Moreover, the mechanism of METTL3-involved tumorigenesis was also explored in this study. Our study may provide molecular insight into enhancing cisplatin sensitivity in cervical cancer cells.

## Results

### METTL3 is frequently downregulated in cervical cancer tissues and cells

**Figure 1:**
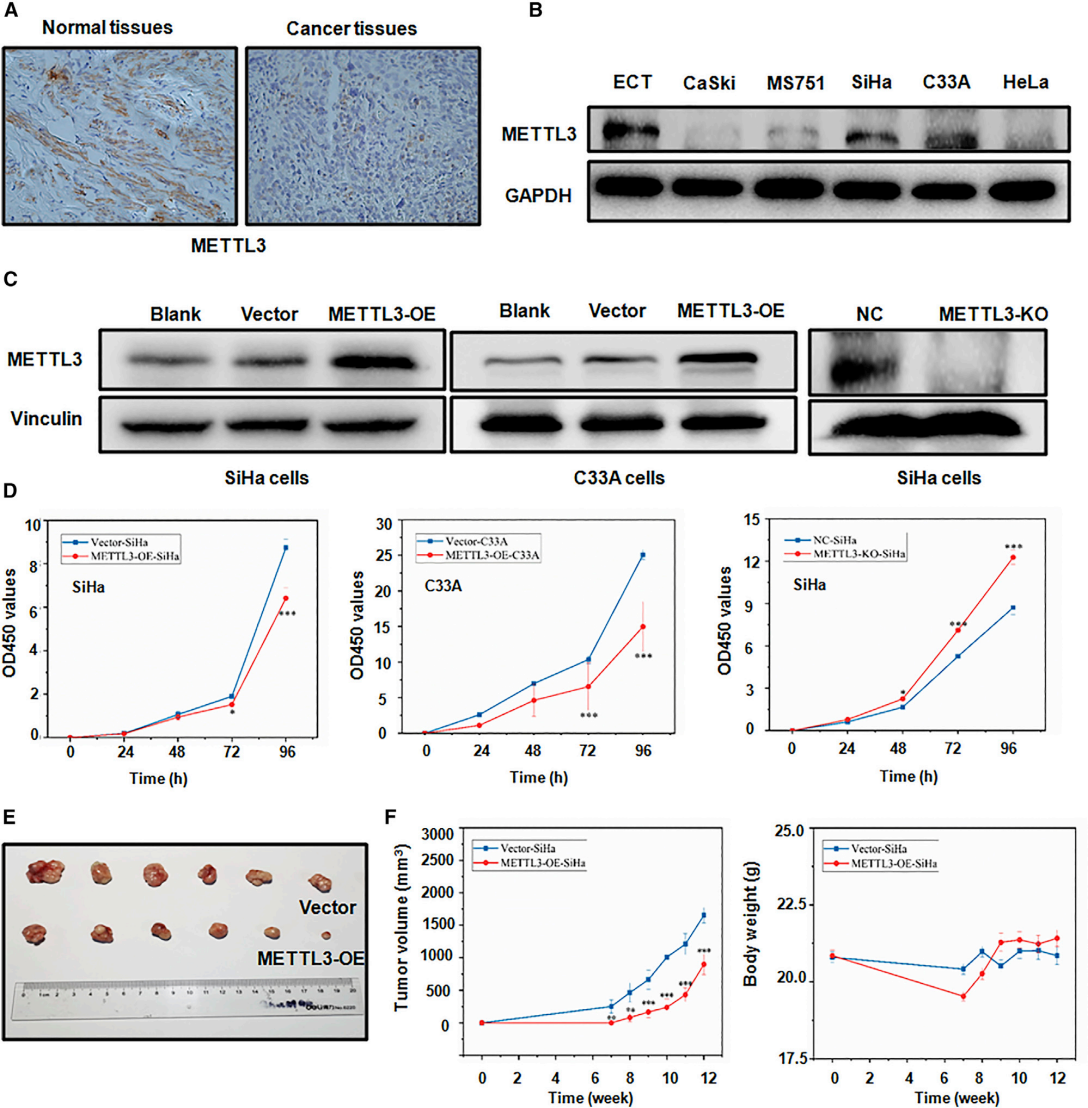
METTL3 overexpression inhibits viability and tumor growth (A) Immunohistochemistry of METTL3 in cervical cancer tissues (original magnification, ×100). (B) METTL3 protein levels in different cervical cancer cells were detected by western blotting. (C) METTL3 protein expression was detected in SiHa cells and C33A cells after METTL3 overexpression or knockout. (D) The cell viability in cells with METTL3 overexpression or knockout was analyzed by a CCK-8 assay. ∗p < 0.05, ∗∗∗p < 0.001 versus vector or NC group. (E) Overexpression of METTL3 inhibited tumor growth. (F) Tumor volumes were measured and calculated (left panel). Body weights were measured and calculated (right panel). ∗∗p < 0.01, ∗∗∗p < 0.001 versus vector group. KO, knockout; NC, negative control; OE, overexpression.

To understand the expression pattern of METTL3 in cervical cancer, an immunohistochemistry (IHC) assay and western blotting analysis were conducted to detect METTL3 protein level in cervical cancer tissues and cell lines, respectively. IHC results showed that METTL3 was significantly downregulated in cervical cancer specimens, whereas the METTL3 protein was mainly localized in the cytoplasm of normal adjacent cervix (Figure 1A). In addition, as compared to the ECT cell line, the expression level of METTL3 was significantly decreased in multiple cell lines of cervical cancer, except in the C33A cell line (Figure 1B). These data demonstrated that METTL3 expression is downregulated in cervical cancer cells and tissues.

### METTL3 inhibits the viability and proliferation of cervical cancer cells *in vitro* and *in vivo*

Among the five cervical cancer cell lines, SiHa and C33A cells displayed the highest expression levels of METTL3 (Figure 1B). Therefore, METTL3 cDNA was stably transfected into SiHa and C33A cells after lentivirus transfection (Figure S1). In addition, METTL3 was knocked out in SiHa cells using CRISPR-Cas9-mediated gene editing. The efficacies of METTL3 overexpression and knockout were confirmed by western blotting (Figure 1C). A Cell Counting Kit-8 (CCK-8) assay was performed to measure the viability of cervical cancer cells after METTL3 modulation. We found that overexpression of METTL3 in SiHa and C33A cells significantly inhibited cell viability compared with their control cells (Figure 1D; p < 0.001). Conversely, SiHa cells with METTL3 knockout (METTL3-KO-SiHa) displayed markedly increased cell viability as compared with control cells (Figure 1D; p < 0.001).

To explore the biological function of METTL3 *in vivo*, a tumor xenograft model was established. A total of 5 × 10^6^ METTL3-overexpressing SiHa cells (METTL3-OE-SiHa) and control cells (vector-SiHa) were injected subcutaneously into the nude mice. As shown in Figure 1E, the tumor xenograft was developed at week 7 after subcutaneous injection. It was clear that the tumors developing from METTL3-OE-SiHa cells grew more slowly than those from vector-SiHa cells from week 7 onward (Figure 1F; p < 0.001). The body weights of mice in each group were not significantly different (Figure 1F). These results collectively indicated that METTL3 could inhibit the viability and tumor growth of cervical cancer cells both *in vitro* and *in vivo*.

### METTL3 increases cisplatin chemosensitivity of cervical cancer cells *in vitro*

**Figure 2:**
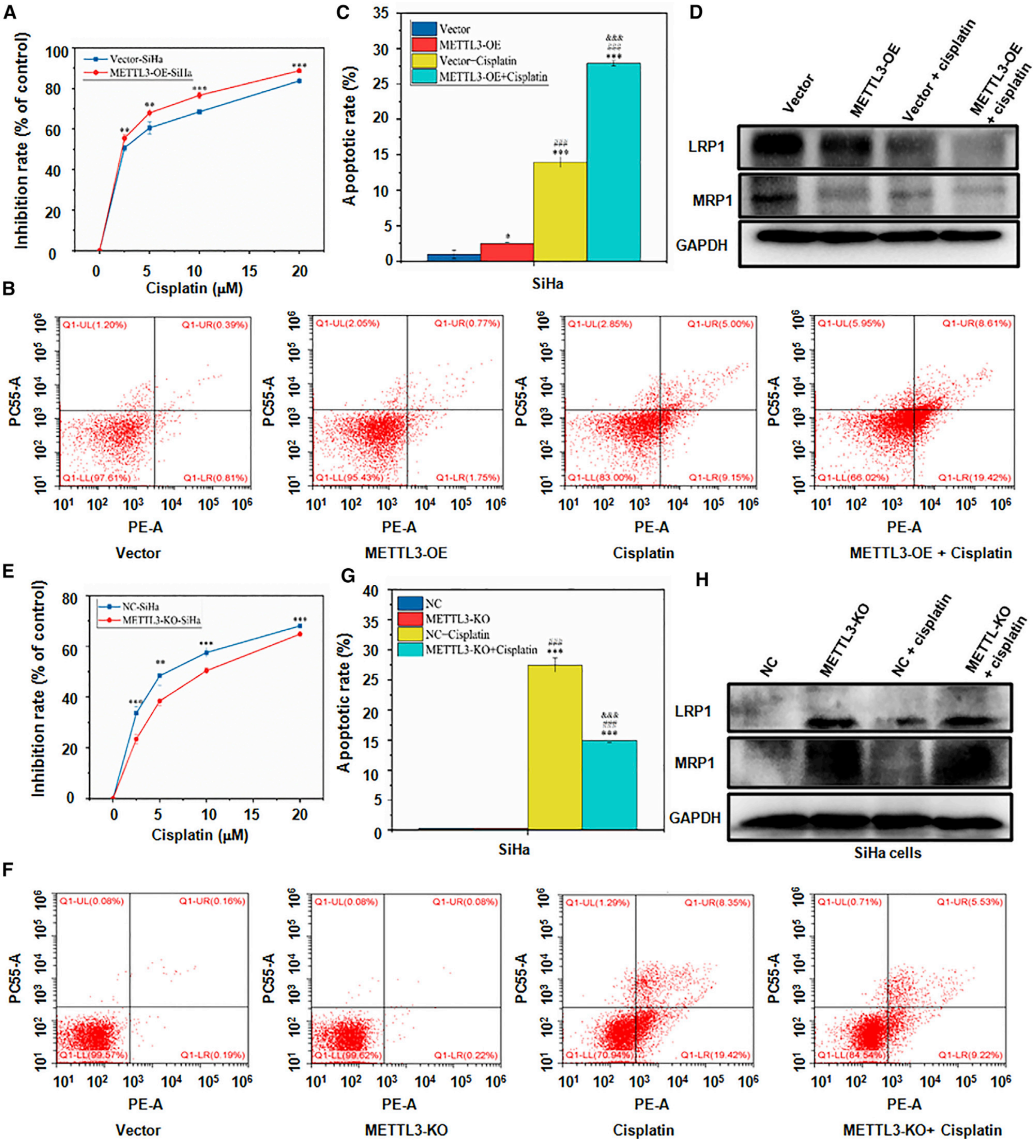
The effect of METTL3 on cisplatin sensitivity in cervical cancer cells (A) The difference of chemotherapeutic sensitivity of cisplatin between SiHa cells with METTL3 overexpression and vector-SiHa cells was analyzed by a CCK-8 assay. ∗∗p < 0.01, ∗∗∗p < 0.001 versus vector group. (B and C) The difference of cisplatin sensitivity between METTL3-overexpressing SiHa cells and control vector-SiHa cells was analyzed by flow cytometry (B). Quantitative results of panel B were shown (C). ∗p < 0.05, ∗∗∗p < 0.001 versus vector group; ^###^p < 0.001 versus METTL3-OE group; ^&&&^p < 0.001 versus vector + cisplatin group. (D) The expression of drug resistance-related proteins LRP1 and MRP1 was detected in METTL3-overexpressing SiHa cells by western blotting. (E) The difference of chemotherapeutic sensitivity of cisplatin between SiHa cells with METTL3 knockout and NC-SiHa cells was analyzed by a CCK-8 assay. ∗∗p < 0.01, ∗∗∗p < 0.001 versus NC group. (F and G) The difference of chemotherapeutic sensitivity of cisplatin between SiHa cells with METTL3 knockout and NC-SiHa cells was analyzed by flow cytometry (F). Quantitative results of panel F were shown (G). ∗∗∗p < 0.001 versus NC group; ^###^p < 0.001 versus METTL3-KO group; ^&&&^p < 0.001 versus NC + cisplatin group. (H) The expression of LRP1 and MRP1 in SiHa cells with METTL3 knockout was detected by western blotting.

To investigate the effect of METTL3 on cisplatin sensitivity of cervical cancer cells, CCK-8 and flow cytometry assays were used under different concentrations of cisplatin (0, 5, 10, 15, and 20 μM). The inhibitory rate in METTL3-OE-SiHa cells was dose-dependently increased compared with that in vector-SiHa cells (Figure 2A; p < 0.001). In addition, upregulation of METTL3 contributed to a significant increase of the apoptotic rate in SiHa and C33A cells (Figures 2B and 2C; Figures S2A and S2B; p < 0.001). Mechanistically, the expression of drug resistance-related proteins MRP1 and LRP1 was evaluated by western blotting analysis. METTL3-OE-SiHa cells showed remarkably less expression of MRP1 and LRP1 and weaker levels under cisplatin administration (Figure 2D; Figure S2C; p < 0.001). Consistently, the cisplatin sensitivity in METTL3-KO-SiHa cells was significantly decreased, as manifested by the reduced cell viability (Figure 2E; p < 0.001) and apoptosis (Figures 2F and 2G; p < 0.001) as well as upregulation of MRP1 and LRP1 (Figure 2H; Figure S2D; p < 0.001). Taken together, these data indicated that METTL3 could increase the sensitivity to cisplatin in cervical cancer cells.

### METTL3 downregulates RAGE expression in cervical cancer tissues and cells

**Figure 3:**
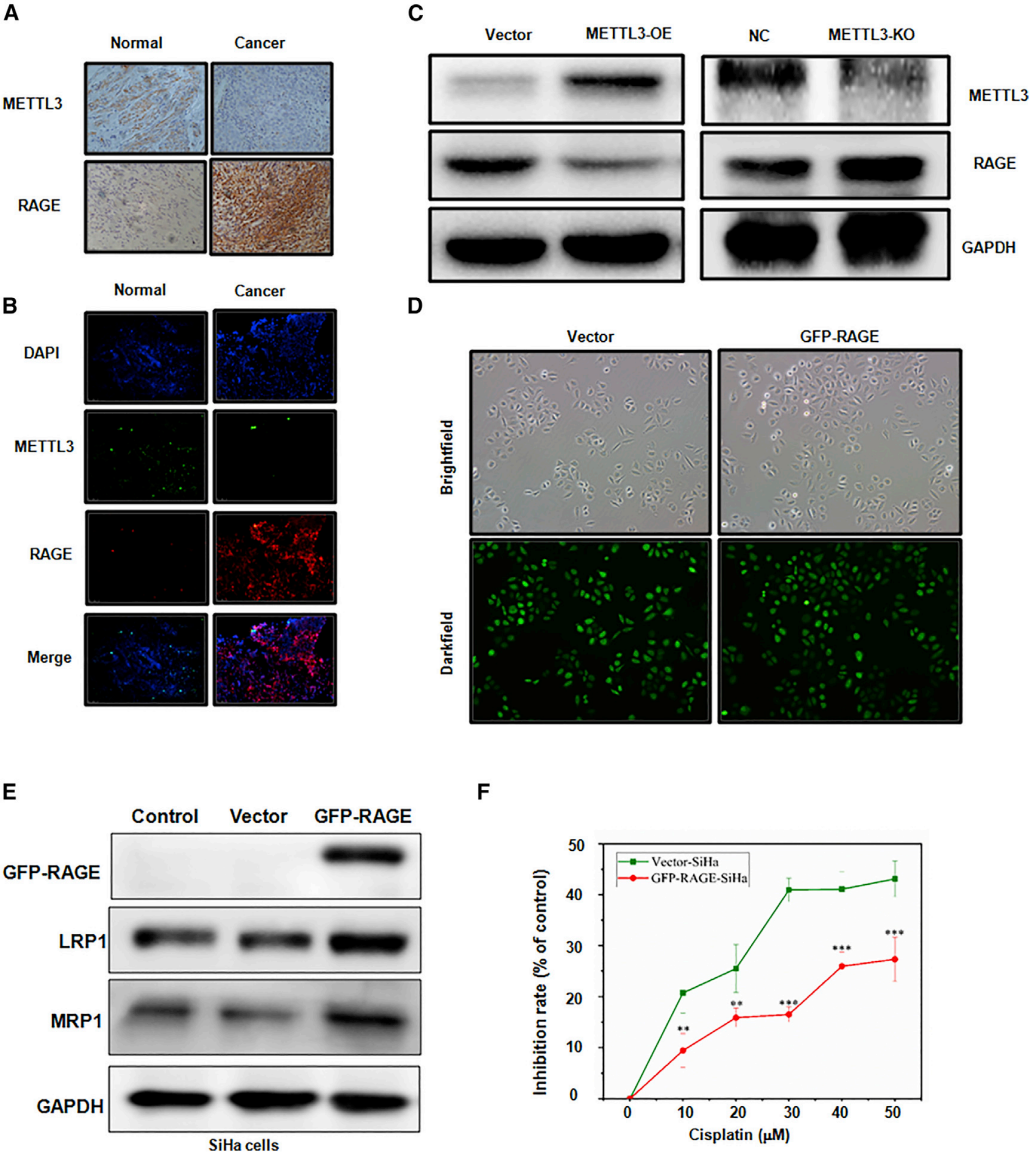
METTL3 regulates RAGE expression in cervical cancer (A) Immunohistochemistry of METTL3 and RAGE in cervical cancer tissues (original magnification, ×100). (B) Immunofluorescence double staining of METTL3 and RAGE in cervical cancer tissues (original magnification, ×100). (C) The expression of METTL3 and RAGE in SiHa cells after METTL3 modulation was detected by western blotting. (D) SiHa cells with RAGE overexpression were observed under the bright field of a microscope, and their fluorescence (GFP) was observed under the dark field of a microscope (original magnification, ×100). (E) The expression levels of LRP1 and MRP1 were detected in RAGE-overexpressing SiHa cells by western blotting. (F) Chemotherapeutic sensitivity of cisplatin in SiHa cells with RAGE overexpression was analyzed by a CCK-8 assay. ∗∗p < 0.01, ∗∗∗p < 0.001 versus vector group.

RAGE has been identified as an oncoprotein to promote cell proliferation and inhibit the apoptosis of cervical cancer cells, suggesting its potential role in regulating cisplatin sensitivity of cervical cancer cells. Therefore, we determined whether METTL3 could target the expression of RAGE in cervical cancer cells, leading to the regulation of cisplatin resistance. To explore the correlation between METTL3 and RAGE in cervical cancer, IHC, immunofluorescence staining, and western blotting analysis were performed. Tumor tissues from cervical cancer showed a nearly negative METTL3 staining but an intense positive staining of RAGE, which was opposite to those in the normal adjacent tissues (Figures 3A and 3B). Additionally, METTL3 overexpression in SiHa cells led to a significant decrease of RAGE expression, while RAGE was upregulated when METTL3 was knocked out (Figure 3C). Taken together, METTL3 could suppress the expression of METTL3 in cervical cancer cells.

### RAGE decreases chemotherapeutic sensitivity of cisplatin in cervical cancer cells

**Figure 4:**
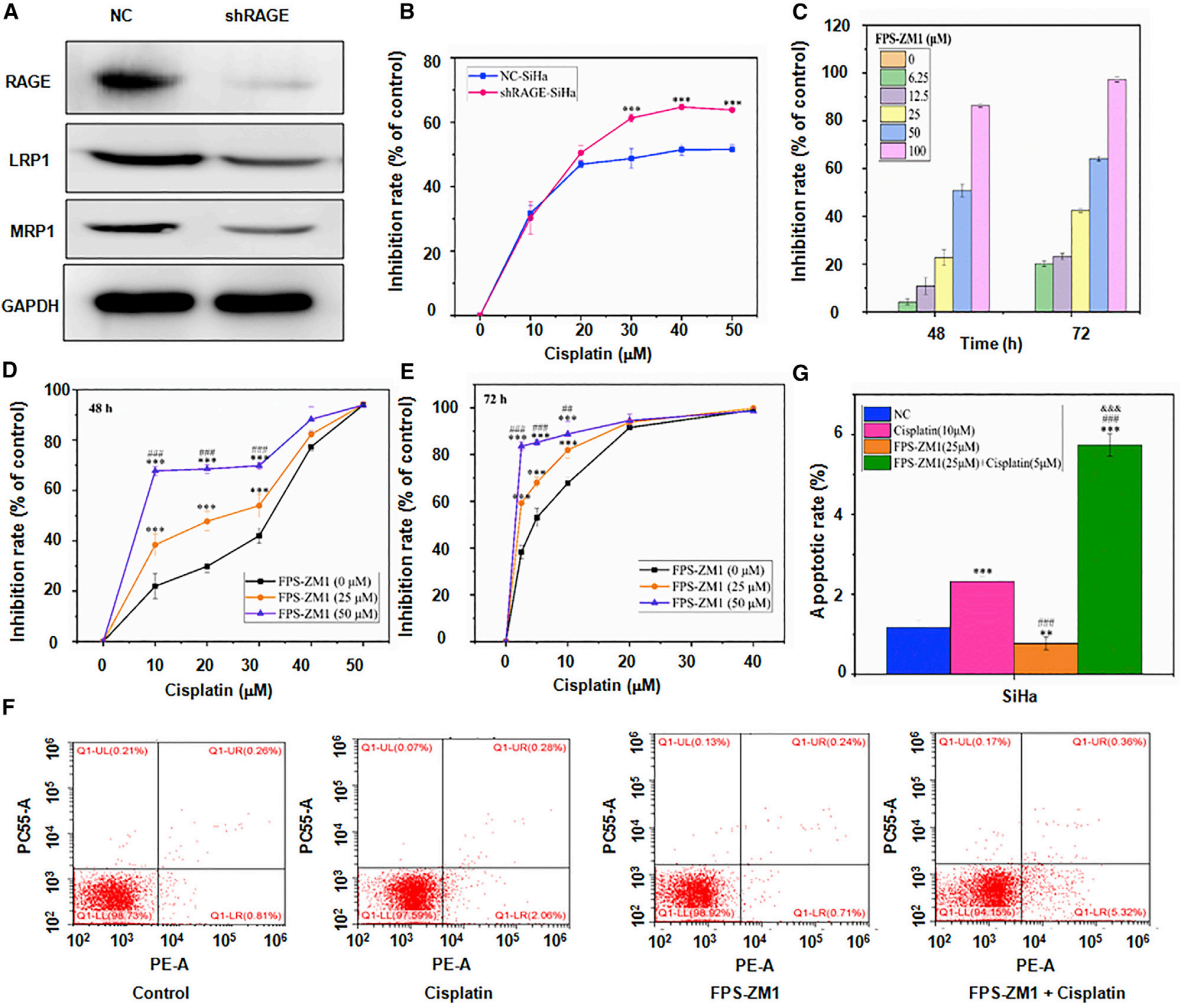
The effects of RAGE on cisplatin sensitivity in cervical cancer cells (A) The expression levels of RAGE, LRP1, and MRP1 proteins in SiHa cells with RAGE downregulation were detected by western blotting. (B) The chemotherapeutic sensitivity of cisplatin in SiHa cells with RAGE downregulation was analyzed by a CCK-8 assay. ∗∗∗p < 0.001 versus vector group. (C) Cytotoxic effect of FPS-ZM1 on SiHa cells was analyzed by a CCK-8 assay. (D and E) Effects of FPS-ZM treatments on cisplatin sensitivity in SiHa cells at 48 (D) and 72 h (E) were performed by a CCK-8 assay. ∗∗∗p < 0.001 versus 0 μM group; ^##^p < 0.01, ^###^p < 0.001 versus 25 μM group. (F and G) Apoptosis rates of SiHa cells after FPS-ZM1 in combination with cisplatin were analyzed by flow cytometry (F). Quantitative results of panel F were shown (G). ∗∗∗p < 0.001 versus NC group; ^###^p < 0.001 versus cisplatin (10 μM) group; ^&&&^p < 0.001 versus FPS-ZM1 (25 μM) group.

To explore effects of RAGE on cervical cancer cells, RAGE expression was upregulated and downregulated by transfecting GFP-conjugated plasmids (Figures 3D and 3E) and RAGE knockdown by short hairpin RNA (shRNA) (shRAGE) (Figure 4A) in SiHa cells, respectively. A significantly dose-dependent decrease of the inhibition rate was observed in GRP-RAGE-SiHa cells (Figure 3F; p < 0.001), whereas shRAGE-SiHa cells exhibited a reduced viability after cisplatin use (Figure 4B; p < 0.001). Moreover, the MRP1 and LRP1 proteins expressed by GFP-RAGE-SiHa cells showed higher levels than those expressed by vector or control cells (Figure 3E; p < 0.001), which was reversed by shRAGE transfection in SiHa cells (Figure 4A). In addition to RAGE knockdown by shRNA, FPS-ZM1, the specific inhibitor of RAGE, was applied to explore the function of RAGE in regulating cisplatin sensitivity in SiHa cells. As shown in Figure 4C, use of 50 μM FPS-ZM1 generated approximately 50% and 60% of the cytotoxic effect on SiHa cells at 48 and 72 h, respectively. Hence, FPS-ZM1 was used at three different concentrations (0, 25, and 50 μM) in the subsequent experiments. Similar results were found in both CCK-8 and flow cytometry analyses, as evidenced by increased viability inhibition and apoptosis rates in a dose-dependent manner (Figures 4D–4G; p < 0.001). Furthermore, the combination of FPS-ZM1 and cisplatin was more effective in inducing cell apoptosis as compared with FPS-ZM1 or cisplatin only (Figures 4F and 4G; p < 0.001). Therefore, these findings indicated that RAGE may be closely related to the sensitivity of cervical cells to cisplatin treatment.

### METTL3 increases cisplatin sensitivity via downregulating RAGE in cervical cancer cells

**Figure 5:**
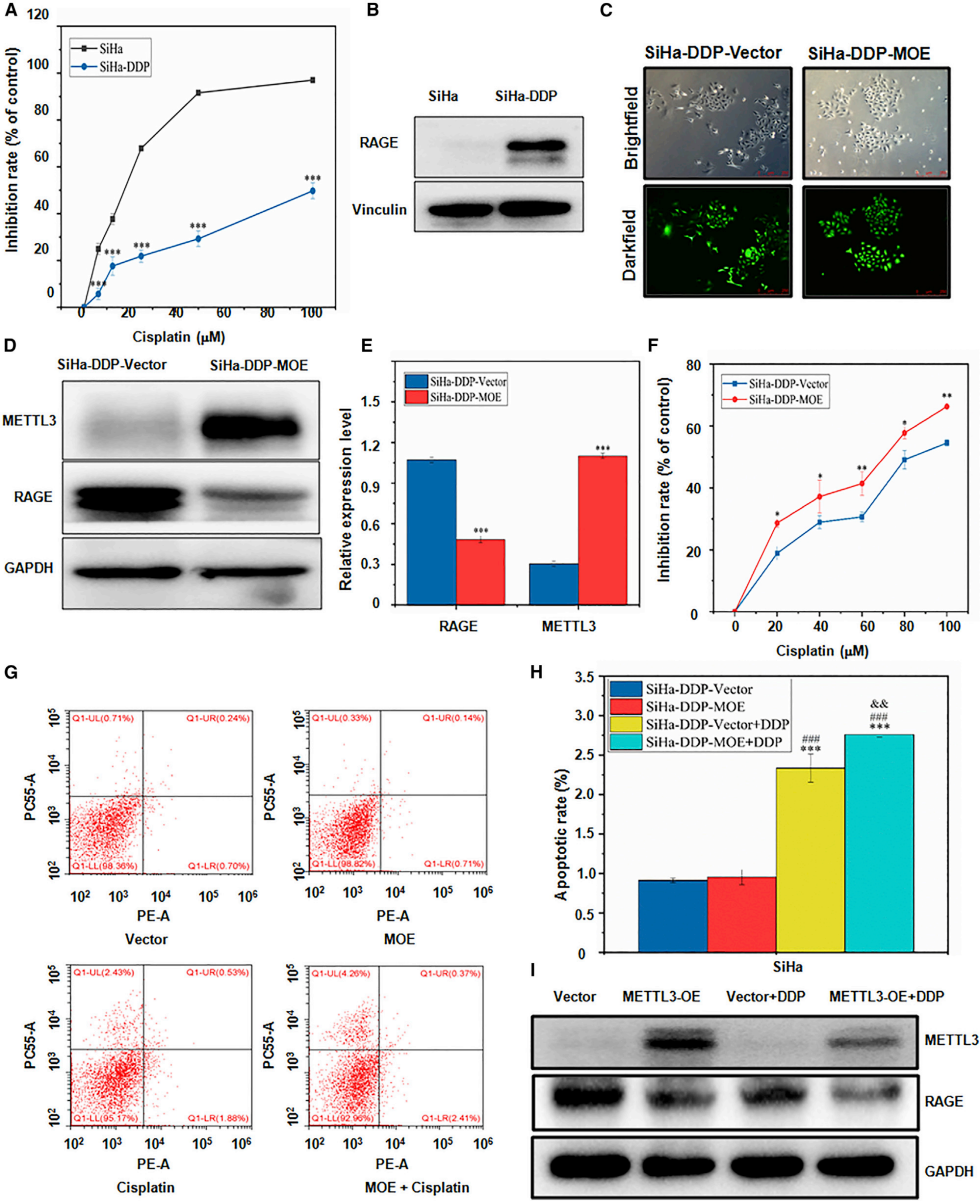
METTL3 overexpression inhibits RAGE protein level in SiHa-DDP cell (A) A CCK-8 assay was used to measure the cisplatin sensitivity in SiHa-DDP cells. ∗∗∗p < 0.001 versus SiHa group. (B) The expression level of RAGE proteins in SiHa-DDP cells was detected by western blotting. (C) The SiHa-DDP cell line with METTL3 overexpression was observed under the bright field of a microscope and fluorescence (GFP) was observed under the dark field of a microscope (original magnification, ×100). (D and E) The expression of METTL3 and RAGE proteins in SiHa-DDP cells after METTL3 cDNA transfection was detected by western blotting (D). Quantitative results of panel D were shown (E)∗∗∗p < 0.001 versus vector group. (F) Cisplatin sensitivity in SiHa-DDP cells after METTL3 overexpression was analyzed by a CCK-8 assay. ∗p < 0.05, ∗∗p < 0.01 versus vector group. (G and H) The apoptosis rate was analyzed in SiHa-DDP cells treated with METTL3 cDNA transfection and cisplatin by flow cytometry (G). Quantitative results of panel G were shown (H). ∗∗∗p < 0.001 versus vector group; ^###^p < 0.001 versus MOE group; ^&&^p < 0.01 versus vector + DDP group. (I) The expression levels of METTL3 and RAGE proteins in SiHa cells after METTL3 cDNA transfection and treatment with cisplatin were detected by western blotting. MOE, METTL3 overexpression.

To further validate whether METTL3 increased cisplatin sensitivity in cervical cancer cells, SiHa-cisplatin (DDP) cell lines were obtained. We observed that the inhibitory effect of cisplatin was significantly reduced in SiHa-DDP cells as compared with control SiHa cells (Figure 5A; p < 0.001). Furthermore, western blotting analysis confirmed that RAGE was dramatically upregulated in SiHa-DDP cells (Figure 5B). Next, SiHa-DDP cells were transfected with lentivirus-mediated plasmids (Figure 5C), and METTL3 was upregulated, which was confirmed by western blotting analysis (Figure 5D). Consistently, a pair of opposite expression patterns of METTL3 and RAGE were also detected in SiHa-DDP-METTL3 overexpression (MOE) cells (Figures 5D and 5E; p < 0.001). As compared to SiHa-DDP-vector cells, the cytotoxic function of cisplatin was significantly enhanced in SiHa-DDP-MOE cells (Figure 5F; p < 0.01). Despite no alteration of apoptotic rate in SiHa-DDP-MOE cells, the additional use of cisplatin further promoted the apoptosis of SiHa-DDP-MOE cells (Figures 5G and 5H; p < 0.001). Strikingly, administration of cisplatin in METTL3-OE-SiHa cells induced more significant downregulation of RAGE than that in vector-SiHa cells (Figure 5I). Taken together, these results strongly supported that METTL3 could increase the sensitivity to cisplatin via downregulating RAGE in cervical cancer cells.

## Discussion

METTL3 has been reported to be involved in numerous biological processes, including cell cycle progression, proliferation, apoptosis, migration, and invasion.^21^ Most recently, METTL3 has emerged as an attractive therapeutic target for treatment in different types of human cancer.^22^ Herein, we reported that METTL3 inhibited viability and induced apoptosis of cervical cancer cells and increased the cisplatin sensitivity via downregulating RAGE.

In this study, the expression of METTL3 was first discovered to be downregulated in both cervical cancer tissues and cell lines by IHC and western blotting analyses, respectively. To further elucidate this role of METTL3 in cervical cancer, METTL3 was overexpressed in SiHa and C33A cells, which led to a significant decrease in the viability of cervical cancer cells, while knockdown of METTL3 promoted growth of cervical cancer cells both *in vitro* and *in vivo*. These findings indicate that METTL3 may play a suppressive function on cervical tumorigenesis. Interestingly, there have been several studies concerning the role of METTL3 as an oncoprotein in cervical cancer.^9^^,^^11^^,^^12^ One study revealed that elevated expression of METTL3 is correlated with poor prognosis of cervical cancer patients.^12^ Two other studies demonstrated that METTL3 can enhance the growth of cervical cancer cells and promote tumorigenesis.^9^^,^^11^ Therefore, further investigation is required to determine the role of METTL3 in cervical cancer.

Chemotherapy resistance is a major reason for treatment failure and disease aggravation in cancer patients. Upregulation of METTL3 in SiHa cells inhibited the viability and promoted apoptosis under cisplatin use. In addition, the drug resistant-related proteins MRP1 and LRP1 were also downregulated by METTL3 overexpression, especially when cisplatin was added. Conversely, knockout of METTL3 contributed to a decreased sensitivity to cisplatin in SiHa cells. Therefore, METTL3 is an important promoting regulator of cisplatin sensitivity for cervical cancer. Our previous study reported that RAGE could promote cell proliferation and inhibit apoptosis in cervical cancer,^20^ indicating its potential role in regulating cisplatin sensitivity of cervical cancer cells. In the current study, upregulation of RAGE alleviated the cytotoxic effects of cisplatin on SiHa cells and induced the expression of MRP1 and LRP1, which were reversed by RAGE knockdown. More importantly, use of the specific RAGE inhibitor FPS-ZM1 also decreased cell viability and increased the apoptotic rate of SiHa cells under cisplatin use. These results together suggest that RAGE can decrease the cisplatin sensitivity in cervical cancer cells.

To further understand whether RAGE could be regulated by METTL3 in cervical cancer cells, the clinical relevance of METTL3 and RAGE was established by immunofluorescence and IHC staining. Our results showed that METTL3 and RAGE were oppositely expressed in cervical cancer tissues, indicating the regulatory network between them. Multiple mechanisms in which METTL3 is involved to regulate drug resistance have been reported. For example, METTL3 regulated cisplatin resistance of non-small cell lung cancer via activation of YAP or cell autophagy.^23^^,^^24^ In addition, METTL3 is associated with drug resistance of seminoma and pancreatic cancer.^25^^,^^26^ We found that METTL3 upregulation markedly enhanced the cisplatin sensitivity in SiHa-DDP cells with overexpression of RAGE. Based on these results, a model is strongly supported and proposed that METTL3 regulates cell sensitivity to cisplatin in cervical cancer possibly via inhibition of RAGE.

In conclusion, our study demonstrates that METTL3 suppressed the activity of RAGE, thus increasing cisplatin sensitivity in cervical cancer. However, several questions need to be addressed to fully understand the role of METTL3 in cervical cancer. What are the molecular mechanisms by which METTL3 regulates the expression of RAGE? Could METTL3 increase sensitivity of other chemotherapeutic drugs in cancer patients? What are the other key downstream targets of METTL3? In addition, potential agonists of METTL3 together with RAGE inhibitors should be explored and developed to treat cervical cancer in the future.

## Materials and methods

### Cell lines and culture

Five human cervical cancer cell lines (SiHa, HeLa, MS751, CaSki, and C33A) as well as human normal cervical epithelial cell lines (ECT) were obtained from the Shanghai Cell Biology Medical Research Institute, Chinese Academy of Sciences. Among the five cervical cancer cell lines, C33A cells are human papillomavirus (HPV)-negative and other four cell lines are HPV-positive. In addition, HeLa cells are derived from adenocarcinoma and other four cell lines are derived from squamous cell carcinoma. SiHa-DDP cells were purchased from Hunan Fenghui Biotechnology (Fenghui, China). All cell lines were maintained as a monolayer in Dulbecco’s modified Eagle’s medium (DMEM) supplemented with 10% fetal bovine serum (FBS; Gibco, Thermo Fisher Scientific) and 100 μg/mL penicillin-streptomycin solution (Invitrogen, UK). The cells were incubated at 37°C in a humidified atmosphere with 5% CO_2_.

### Construction of lentivirus and cell transfection

Human METTL3 and GFP-RAGE cDNAs were subcloned into pLenti-C-mGFP vector (Origene, MD, USA) and pCDH-GFP-puro. shRAGE in a pLKO.1-TRC vector (Origene, MD, USA) was used to deplete RAGE. CRISPR-Cas9 was used to deplete METTL3. The pLenti-C-mGFP vector or pLKO.1-TRC vector was used as an empty vector control. Cells treated with Lipofectamine 2000 were used as a blank control. After confirmation by gene sequencing, the virus was packed using 293T cells with two packaging vectors, psPAX2 and pMD2.G. Lentiviral particles were harvested and filtered to infect cervical cancer cell lines.

### Cell viability assay

SiHa or C33A cells (1 × 10^3^ cells/well) were cultured in 96-well plates in 100 μL of culture medium overnight. CCK-8 reagent (10 μL/well, Dojindo, Japan) was added, and they were incubated for another 2 h with 5% CO_2_ at 37°C. We observed and recorded the procedures of cell growth at 0, 24, 48, 72, and 96 h. Cell viability was determined at 450 nm and is represented as a percentage of the control using a microplate reader (Thermo Fisher Scientific, USA).

### Western blotting analysis

The cells were washed with phosphate-buffered saline (PBS) and lysed with radioimmunoprecipitation assay (RIPA) buffer (150 mM NaCl, 1.0% Nonidet P-40, 0.5% sodium deoxycholate, 0.1% sodium dodecyl sulfate [SDS], 50 mM Tris [pH 8.0]) containing protease inhibitor cocktail (Beyotime, Shanghai, China). The lysates were incubated on ice for 30 min. After centrifugation at 12,000 × *g* at 4°C for 20 min, the supernatant was collected and a bicinchoninic acid (BCA) assay (Beyotime, China) was used for protein qualification. Equal amounts of proteins were loaded onto a 10% or 12% SDS-polyacrylamide gel electrophoresis (PAGE) and then transferred onto a polyvinylidene fluoride (PVDF) membrane (Millipore, Boston, MA, USA). The membranes were blocked with 5% non-fat milk in Tris-buffered saline containing 0.1% Tween 20 at room temperature for 1.5 h, and incubated at 4°C overnight with each primary antibody as follows: MRP1 and LRP1 (1:1,000, Abcam, USA), METTL3 (1:500) (Abcam, San Francisco, CA, USA), GFP (1:1,000, Cell Signaling Technology, Beverly, MA, USA), vinculin (1:1,000, Invitrogen, UK), RAGE (1:1,000, Santa Cruz, USA), and GAPDH (1:5,000, Weiao, Shanghai, China). Then, the membranes were incubated with the secondary antibody for 1.5 h at room temperature. Enhanced chemiluminescence reagent (Beyotime, China) was used to detect the protein signals. All experiments were performed in triplicate.

### Analysis of cell sensitivity to cisplatin

The viability of the SiHa cells after treatment with cisplatin (Sigma-Aldrich, USA) was determined using the CCK-8 assay. The cells were digested and cultured in 96-well plates for 24 h. Subsequently, the cells were treated with various concentrations of cisplatin (0, 10, 20, 30, 40, 50, 60, 70, and 80 μM) for 48 h or concentrations of cisplatin (0, 2.5, 5, 10, and 20 μM) for 96 h. Then, the drug solution was replaced with fresh medium, and 10 μL/well CCK-8 solution was added to the medium. Cells were incubated at 37°C for 2 h, and absorbance measured at 450 nm absorption spectra in a microplate reader (Thermo Fisher Scientific, USA). The inhibition rate was calculated as follows: cell viability (%) = [(optical density [OD]_450_ of control well – OD_450_ of test well)/(OD_450_ of control well – OD_450_ of blank well)] × 100%. The experiments were repeated three times.

### Flow cytometry analysis

Cells were plated at 5 × 10^5^ cells/dish into 60-mm dishes. After appropriate treatment and incubation, cells were harvested, washed with cold PBS, and resuspended with binding buffer at a concentration of 1 × 10^6^ cells/mL. Then, the cells were double-stained with phycoerythrin (PE)/7-aminoactinomycin D (7-AAD) according to the manufacturer’s protocol (BD Pharmingen, CA, USA). The percentage of apoptotic cells was detected by flow cytometry after staining. The experiment was repeated three times.

### IHC assay

The approval of this study was obtained from the Ethics Committee of The Second Affiliated Hospital of Wenzhou Medical University. After fixation, samples were paraffin embedded and sliced. The sections were microwaved for antigen retrieval and incubated with RAGE monoclonal antibody (mAb; diluted at a 1:50 ratio, Santa Cruz, USA) or METTL3 mAb (diluted at a 1:200, Abcam, USA) overnight at 4°C. Then, the sections were washed with PBS, incubated with goat anti-mouse or goat anti-rabbit secondary antibodies for 20 min, and a chromogenic reaction was performed at room temperature using a diaminobenzidine (DAB) substrate kit (Sigma, USA) for color development. Then, sections were counterstained with hematoxylin. Representative images of tissues were captured using a microscope (Leica Microsystems, Bannockburn, IL, USA).

### Immunofluorescence staining

Cervical cancer or adjacent non-tumor cervical tissues were embedded in O.C.T. compound (Leica Microsystems, Bannockburn, IL, USA) and 10-μm sections were cut using cryostat. Prior to immunofluorescence staining, slides were fixed with methanol for 20 min. After washing with PBS three times, slides were permeabilized in Triton X-100 for 15 min and incubated with METTL3 (1:500, Abcam) and RAGE (1:50, Abcam) primary antibodies overnight at 4°C. Slides were washed with PBS three times and incubated with secondary antibodies (goat anti-rabbit Alexa Fluor 488 or goat anti-mouse Alexa Fluor 594, 1:200 dilution, Life Technologies) for 1 h. Tissue sections were mounted in Vectashield mounting medium containing 4′,6-diamidino-2-phenylindole (DAPI; Vector Laboratories, Burlingame, CA, USA), imaged with fluorescent microscopy (Leica Microsystems), and prepared by a Zeiss LSM image browser software.

### Tumor xenograft model

Female BALB/c (nu/nu) mice (4–5 weeks of age; Laboratory Animal Resources, Chinese Academy of Sciences) were group-housed under a constant 12-h light/12-h dark cycle and were provided with sterilized food and water. Ethics and legal approvals were obtained prior to the commencement of the animal study. All experimental procedures using live animals were conducted in accordance with protocols approved by the Wenzhou Medical University Institutional Animal Care and Use Committee and national guidelines and regulations. On the basis of a lentivirus system, which can stably upregulate METTL3 in cervical cancer cell lines, xenograft tumors were established by subcutaneously injecting SiHa cells transfected with METTL3 (5 × 10^6^ cells/mouse) in a total volume of 0.1 mL in PBS. Tumor volumes were calculated from caliper measurements. In detail, the long and short diameters of tumors were measured and then calculated by the standard formula where volume = (long diameter × short diameter × short diameter)/2. When tumors grew to 100 mm^3^, cisplatin was injected intraperitoneally, and saline was injected intraperitoneally as control.

### Statistical analysis

All statistical analyses were performed with SPSS 22.0 statistical software (SPSS, Chicago, USA). Data are expressed as mean ± standard deviation. All experiments were performed at least in triplicate. A two-tailed Student’s t test was used to analyze differences between two groups, and a one-way analysis of variance (ANOVA) was used to analyze differences among multiple groups (more than two groups) when the data were normally distributed. A Levene test was used for homogeneity test of variance, and the least significant difference (LSD) was performed for homogeneous data, while a Dunnett’s T3 test was performed for heterogeneous data. p < 0.05 was considered to be statistically significant.

## Supplemental information

Document S1. Figures S1 and S2

Document S2. Article plus supplemental information
