# Supplementary material for: METTL3 increases cisplatin chemosensitivity of cervical cancer cells via downregulation of the activity of RAGE
Source: Mol Ther Oncolytics. 2021 Jun 4;22:245–55. doi: 10.1016/j.omto.2021.05.013 (PMC8424130; doi:10.1016/j.omto.2021.05.013)
Supplement: Document S1. Figures S1 and S2 [file mmc1.pdf]

OMTO, Volume *22*

Supplemental information

METTL3 increases cisplatin chemosensitivity of cervical cancer cells via downregulation of the activity of RAGE

Ruyi Li, Yizuo Song, Xin Chen, Man Chu, Zhi-wei Wang, and Xueqiong Zhu

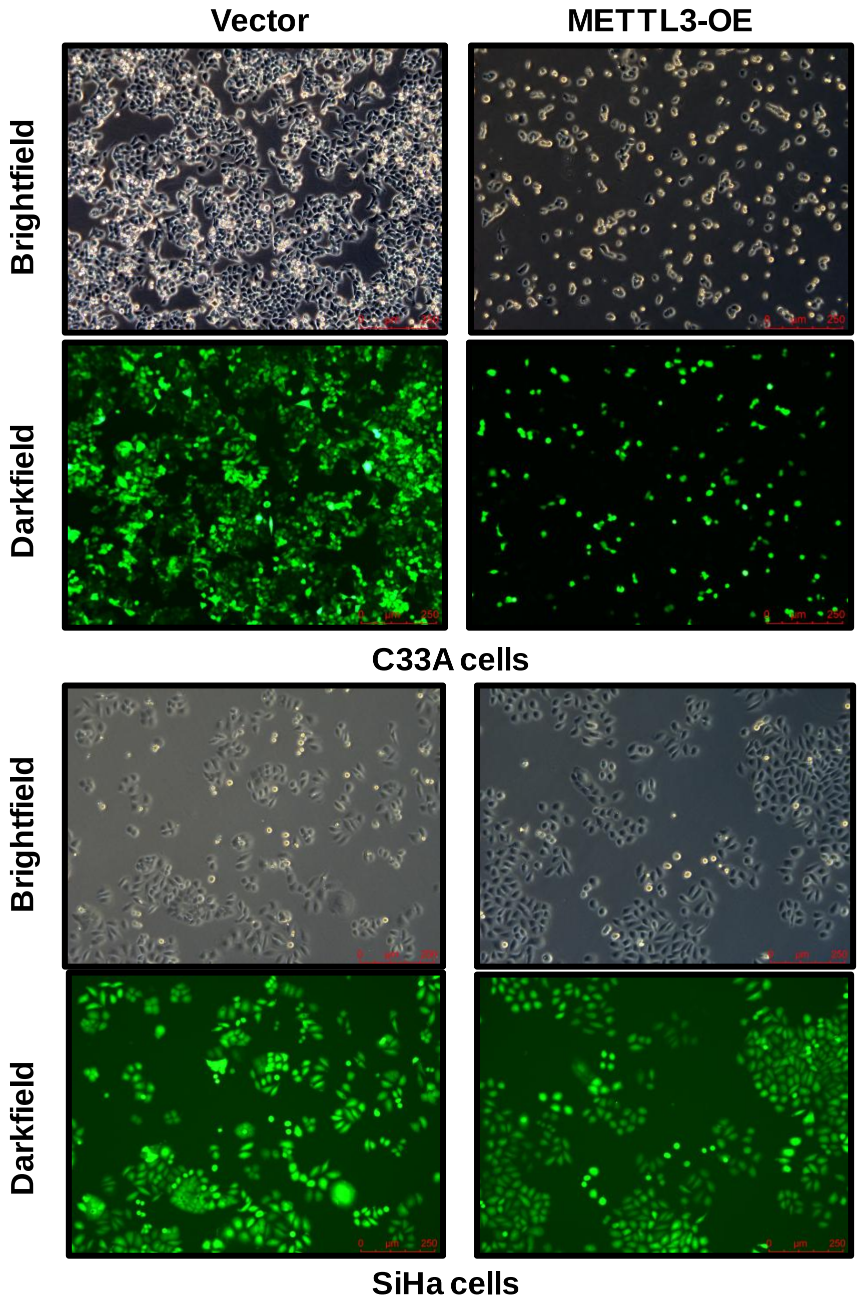

Supplementary figure 1. METTL3 inhibits viability of cervical cancer cells. SiHa cells with METTL3 cDNA transfection was observed under brightfield, and their GFP-green fluorescences were observed under darkfield of microscope (×100 magnification).

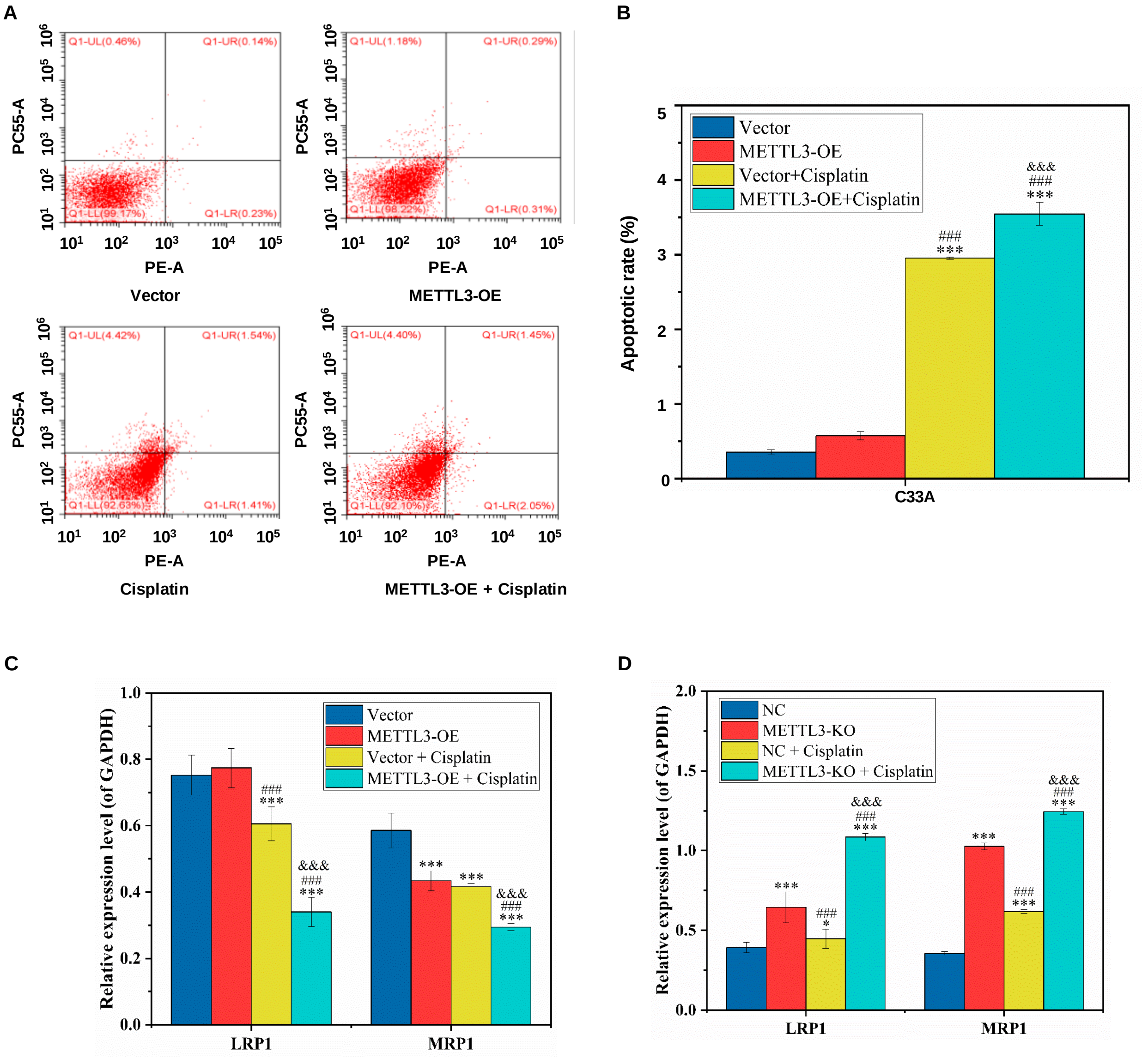

Supplementary figure 2: The effect of METTL3 on cisplatin sensitivity in cervical cancer cells. A-B: The difference of chemotherapeutic sensitivity of cisplatin between C33A cells with METTL3 overexpression and Vector-C33A cells was analyzed by Flow cytometry. C: Quantitative results are shown for Figure 2D. D: Quantitative results are shown for Figure 2H.
